# The Three Glories

**Published:** 1876-04

**Authors:** 


Of our merchant princes, there are two classes, since the financial tornado has swept across the commercial world. Those who withstood its fury walk now with a firmer tread and higher beating hearts; their glory is, that they still stand. Of the fallen, there are those who fell, proud as ever to-day, because they fell honorably. But our wives and daughters revel in a glory that towers a head and shoulders above all others, and throws all others into the shade—the glory of purchasing dry goods at half price.

